# Clinical and Prognostic Differences Between Mechanical Versus Biological Prosthetic Infective Endocarditis—A Nationwide Database Study

**DOI:** 10.3390/jcm14248826

**Published:** 2025-12-13

**Authors:** Juan Esteban de Villarreal-Soto, Jorge Calderón Parra, Patricia Muñoz García, Gregorio Cuerpo Caballero, Marina Machado Vílchez, Maria Ángeles Rodríguez-Esteban, Raquel Rodriguez-Garcia, Valentín Tascon-Quevedo, Ane Josune Goikoetxea-Agirre, Eduard Quintana Obrador, Miguel Angel Goenaga-Sanchez, Elisa Garcia-Vazquez, Rafael Hernandez-Estefania, Antonio Ramos Martínez, Carlos Esteban Martin-López

**Affiliations:** 1 Cardiac Surgery Department, Hospital Universitario Puerta de Hierro Majadahonda, 28222 Majadahonda, Spain; 2 Infectious Diseases Department, Hospital Universitario Puerta de Hierro Majadahonda, 28222 Majadahonda, Spain; jorge050390@gmail.com (J.C.P.); aramos220@gmail.com (A.R.M.); 3 Clinical Microbiology and Infectious Diseases Department, Hospital General Universitario Gregorio Marañón, 28010 Madrid, Spain; 4 Cardiac Surgery Department, Gregorio Marañon University Hospital, 28010 Madrid, Spain; 5 Intensive Care Unit, Hospital Central de Asturias, 33011 Oviedo, Spainrakel_20r@hotmail.com (R.R.-G.); 6 Cardiac Surgery Department, Hospital Universitario Marques de Valdeclla—IDIVAL, 39008 Santander, Spain; 7 Infectious Diseases Department, Hospital Universitario de Cruces, 48903 Bilbao, Spain; 8 Cardiovascular Surgery Department, Hospital Clínic of Barcelona, 08036 Barcelona, Spain; 9 Infectious Diseases Department, Hospital Universitario Donosti, 20014 San Sebastián, Spain; goenagasanchez@gmail.com; 10 Internal Medicine Department, Hospital Clínico Universitario Virgen de la Arrixaca, 30120 Murcia, Spain; 11 Cardiac and Thoracic Surgery Department, Hospital Universitario Fundación Jiménez Díaz, 28040 Madrid, Spain

**Keywords:** endocarditis, valve surgery, mechanical prosthesis, biological prosthesis, registry

## Abstract

**Objectives**: Infective endocarditis (IE) is a feared and life-threatening complication, requiring a multidisciplinary approach. Prosthetic valve endocarditis (PVE) accounts for 20–30% of IE, is one of the most severe forms of IE, and is associated with high morbidity and mortality. We aim to compare and analyze baseline characteristics, microbiology, clinical presentation, complications, and prognosis between biological and mechanical PVE; we also carried out a subgroup analysis of patients aged 45–65 at the time of onset of prosthetic surgery. **Methods**: The present study is a post hoc analysis of a prospective multicenter cohort of patients with PVE between January 2008 and December 2023. Patients were divided into two groups regarding the type of prosthesis, mechanical vs. biological. **Results**: A total of 1544 patients were included. 733 (47.47%) patients with mechanical PVE (mPVE) and 811 (52.52) with biological PVE (bPVE). We found that bPVE appeared earlier than mPVE, had more healthcare-related infections and paravalvular complications. Both groups had similar clinical presentations; moreover, there was no difference in surgical indication and if surgery was performed. On the other hand, mPVE has a higher incidence of *Staphylococcus aureus* (SA) and Gram-negative bacteria, while bPVE has more coagulase-negative staphylococci. Multivariable logistic regression identified the following independent risk factors of mortality: EuroSCORE I, age, mPVE, SA, IE comprising two valves, and severe sepsis. mPVE had a higher mortality on admission, probably due to a higher incidence of septic shock and CNS embolism. The subgroup analysis of patients between 45 and 65 years at the time of prosthesis implantation showed similar results. **Conclusions**: The present analysis shows that bPVE appears earlier than mPVE, even in the subgroup of patients aged 45–65. bPVE has more healthcare-related infections and more paravalvular complications. After adjusting for baseline differences, mPVE had higher in-hospital mortality.

## 1. Introduction

Infective endocarditis (IE) represents a feared and life-threatening complication of increasing prevalence, necessitating a rapid, multidisciplinary approach to achieve favorable outcomes. Prosthetic valve endocarditis (PVE) constitutes one of the most severe forms of IE, currently accounting for 20–30% of all IE cases [1,2]. PVE is consistently associated with alarmingly high morbidity, often requiring complex surgical re-interventions, and maintains high in-hospital mortality rates that range from 22 to 40% [3]. Moreover, retrospective data from the Spanish National Health System [4] highlight a substantial increase in the incidence of PVE, increasing from 10% in the year 2000 to 20% in 2019. This trend is paralleled by an undeniable increase in the clinical complexity of patients requiring operative management for IE [5], where advanced age, extensive comorbidities, and profound structural cardiac destruction are established determinants of poor prognosis and high mortality [5,6].

The evolution of cardiovascular medicine has resulted in significant advancements in diagnostic and therapeutic strategies. The consolidation of multidisciplinary endocarditis teams, refined surgical techniques, the introduction of advanced laboratory assays, and nuclear medicine tools all aim for a more effective and rapid diagnosis, allowing for an individualized and tailored approach for each patient [7]. However, this progress is complicated by concurrent demographic shifts: the aging of the population, the growing preference for biological prostheses in younger patients, and the increased use of transcatheter valves have fundamentally changed the paradigm of IE management. While numerous previous studies have emphasized the critical differences between native valve IE and PVE [8,9,10,11], and more recently between transcatheter and surgical valve IE [12,13,14], there remains a significant knowledge gap detailing the differences in patient characteristics, microbiology, clinical presentation, and prognosis, specifically between biological PVE (bPVE) and mechanical PVE (mPVE). To the best of our knowledge, there is no current study specifically addressing the differences between bPVE and mPVE. This distinction may be crucial due to the differences in surface material, potential for structural failure, and varying infection mechanisms, particularly within working-age adults.

Addressing this critical data scarcity, our study aims to compare bPVE and mPVE, placing emphasis on patient baseline characteristics, microbiology, clinical presentation, complications, and overall prognosis. Recognizing the growing trend of biological valve implantation in middle-aged individuals, a dedicated subgroup analysis of patients aged 45–65 years at the time of prosthetic surgery has been carried out to further elucidate any specific differences in PVE within this important and less-studied age group.

## 2. Materials and Methods

### 2.1. Ethical Statement

The GAMES registry was approved by the regional ethics committee of Madrid (CEIC-R, for its Spanish abbreviation) with code identification ENEI on 11 January 2008. Informed consent was obtained from patients upon inclusion in the registry. Additional ethics committee approval was obtained for this retrospective analysis at the Puerta de Hierro University Hospital ethics committee (code PI 191/24, date of approval 9 December 2024), and an additional individual informed consent was waived.

### 2.2. Study Design

We performed a post hoc analysis of the prospective, multicenter GAMES registry, which encompasses data collected from over 40 centers in Spain between 15 January 2008 and December 2022. The study cohort included patients aged ≥18 years who were diagnosed with prosthetic valve infective endocarditis (PVE). We excluded patients with native valve infective endocarditis, those who had undergone transcatheter valve implantation or repair, and those possessing both biological and mechanical prostheses in different valve positions.

Within the GAMES registry, a multidisciplinary team at each participating center completed a standardized, anonymized form for every IE episode, along with a one-year follow-up form. Collected data included baseline characteristics, microbiology, clinical presentation, management, and outcomes. The registry form also included an evaluation of the theoretical surgical risk using the EuroSCORE I, which was the established metric at the time the registry was created.

### 2.3. Definitions and Patient Groups

Infective endocarditis (IE) was defined according to the modified Duke criteria in effect at the time of each episode [13], and microbiological diagnosis relied on positive blood or valve cultures. Definitions for hospital-acquired, non-healthcare-related, and community-acquired IE followed those established in previous studies [15]. Chronic renal failure was defined as a previous serum creatinine level > 1.4 mg/dL, while worsening or new-onset renal impairment was defined as a deterioration of at least 25% in creatinine clearance, as measured by the Cockcroft–Gault equation. Persistent bacteremia was defined as positive blood cultures persisting for more than seven days after initiating effective antibiotic therapy, and relapses were defined as a new IE episode caused by the same microorganism within the first year of follow-up. Variables were collected to calculate the Charlson Comorbidity Index, and surgical indications were based on the latest European guidelines [13]. Patients who met surgical indications but did not undergo an operation were specifically identified.

Finally, patients were divided into two mutually exclusive groups based on the type of infected prosthesis: mechanical prosthetic valve endocarditis (mPVE) and biological prosthetic valve endocarditis (bPVE).

### 2.4. Statistical Analysis

Categorical variables are expressed as frequencies and percentages as appropriate. Continuous variables are expressed as mean and standard deviation for normally distributed variables and as median and interquartile range for nonnormally distributed variables. Differences between both groups were analyzed using the chi-square test (or exact Fisher test when necessary) for categorical variables and the Mann–Whitney U test for continuous variables. Multivariable logistic regression was used to identify independent risk factors associated with mortality. Odds ratios (ORs) and 95% confidence intervals (95% CI) are provided. A one-year survival time-to-event analysis was performed with Cox multivariable regression. Hazard ratio (HR) and 95% CI are provided. A comparative analysis was carried out for the entire cohort. Additionally, a subgroup analysis was performed on patients between 45 and 65 years old at the time of prosthesis implantation.

Bilateral *p*-values inferior to 0.05 were considered statistically significant. All statistical analyses were performed by means of the SPSS statistical software package (version 25, IBM, Armonk, NY, USA).

## 3. Results

### 3.1. Preoperative and Baseline Characteristics

**Figure 1:**
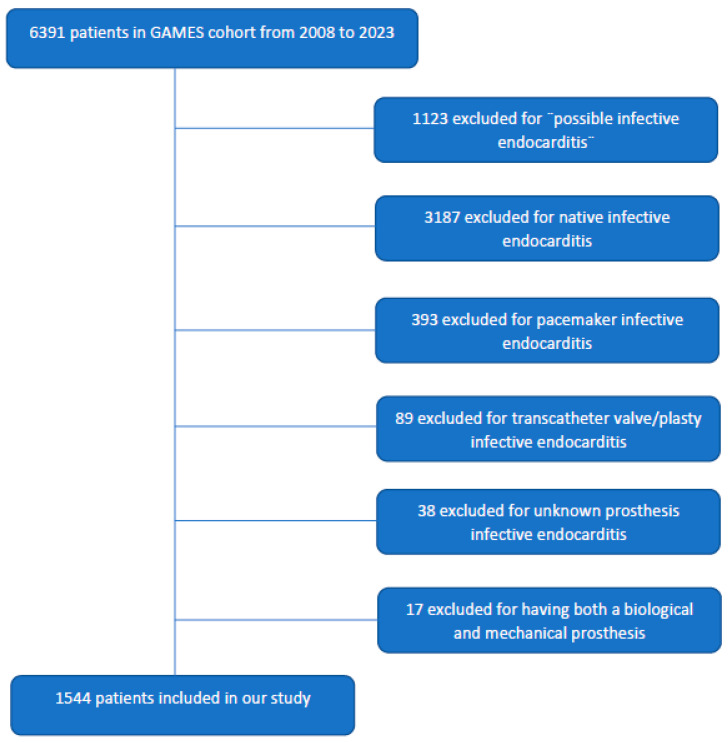
Patient inclusion.

A total of 1544 patients were included in this analysis. 733 (47.47%) patients with mPVE and 811 (52.52) with bPVE (Figure 1). Patient characteristics are described in Table 1.

Although the two groups were generally similar, they exhibited statistically significant differences in several baseline characteristics. Patients in the biological PVE (bPVE) group were significantly older and had a higher incidence of hypertension, chronic obstructive pulmonary disease (COPD), and coronary artery disease (CAD). Consistent with these findings, the bPVE group also had higher scores for the age-adjusted Charlson Comorbidity Index and the EuroSCORE I surgical risk assessment. Conversely, patients in the mechanical PVE (mPVE) group showed a higher incidence of preoperative atrial fibrillation, existing preoperative pacemaker (PM) or implantable cardioverter-defibrillator (ICD), and a history of previous infective endocarditis. No significant differences were observed between the groups for the remaining analyzed variables. Furthermore, the onset of IE was significantly earlier in the bPVE group, occurring at a median of 19 months (interquartile range, p25–p75: 4–51 months) after the index surgery, compared to a median of 56 months (interquartile range: 12–151 months) in the mPVE group.

### 3.2. Microbiological Data

The two prosthetic groups demonstrated statistically significant differences in microbiological profiles and infection acquisition patterns. mPVE was associated with a higher incidence of *Staphylococcus aureus* (SA) (19.9% vs. 11.7%, *p* < 0.001) and Gram-negative bacteria (5.7% vs. 3.5%, *p* = 0.032). Conversely, bPVE showed a higher proportion of coagulase-negative staphylococci (32.8% vs. 26.2%, *p* = 0.005) and *Enterococcus* spp. (17.8% vs. 13.4%, *p* = 0.018). There were no differences found regarding infection by *Streptococci* spp., *Candida* spp., or polymicrobial endocarditis, and the primary focus of infection was similar between the groups. Regarding acquisition, mPVE presented as community-acquired in a higher percentage of cases (61.4% vs. 55.2%, *p* = 0.014), while healthcare-associated infections were significantly more common in the bPVE group (38.2% vs. 32.3%, *p* = 0.016). Microbiological data are summarized in Table 1.

### 3.3. Clinical Presentation

Regarding clinical presentation, there were no significant differences between the two groups concerning fever, persistent bacteremia, central nervous system (CNS) embolism, peripheral embolism, or acute heart failure. However, mPVE patients presented with a statistically higher incidence of severe sepsis at hospital admission (19.9% vs. 14.0%, *p* = 0.002), IE involving two valves (18.7% vs. 13.3%, *p* = 0.004), and prosthetic valve dehiscence (27.6% vs. 18.1%, *p* < 0.001). Conversely, bPVE patients demonstrated a higher incidence of abscess formation (37.3% vs. 31.2%, *p* = 0.012). Notably, there was no difference between the groups in the rate of surgical indication or the actual performance of surgery. Clinical presentation findings are summarized in Table 1.

### 3.4. Outcomes and Postoperative Complications

There were no statistically significant differences in the postoperative complications between the two groups. Postoperative complications are summarized in Table 2.

Unadjusted in-hospital and 1-year mortality were not different between mPVE and bPVE patients (32.5% vs. 30.0%, *p* = 0.288; and 36.8% vs. 35.5%, *p* = 0.589, respectively). Table 3.

**Figure 2:**
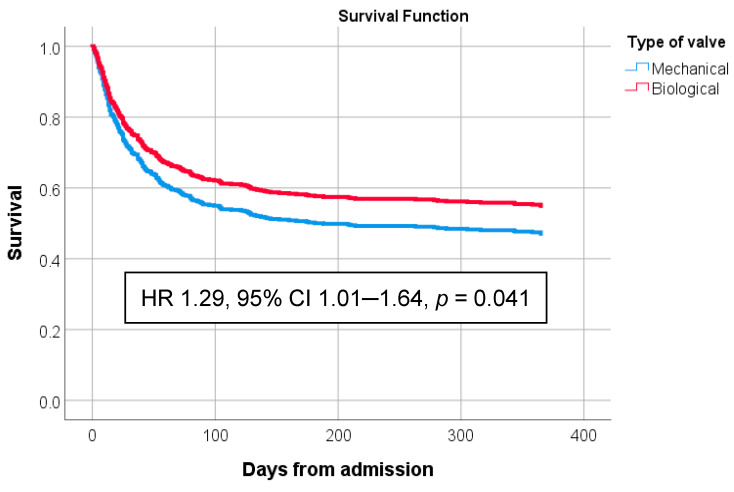
Kaplan–Meier survival curve representing 1-year survival of patients with bPVE and mPVE. The hazard ratio was calculated by means of a one-step multivariate Cox regression model. Variables included in the model were mechanical prosthesis (versus biological), gender, age, Charlson index, *Staphylococcus aureus*, coagulase-negative staphylococci, *Enterococcus* spp., Gram-negative bacilli, aortic valve implication, two or more valve implications, dehiscence, peripheral embolization, central nervous system embolization, septic shock, and EuroSCORE I. spp.: Species.

After adjusting for baseline differences in multivariable regression models, mechanical prosthesis was associated with higher in-hospital mortality (OR 1.48, confidence interval 95% 1.04–2.11, *p* = 0.028). Additionally, mechanical prosthesis was associated with higher 1-year mortality (HR 1.29, 95% confidence interval 1.01–1.64, *p* = 0.041) (Figure 2). Multivariable logistic regression identified independent risk factors associated with mortality: mPVE, age, Charlson index, SA, IE comprising two valves, and septic shock (Table 4).

### 3.5. Differences in Mechanical Versus Biological Endocarditis According to Location of the Prosthesis

Further analysis comparing IE regarding the location of the prosthesis was carried out. Data is summarized in Supplementary Materials. Similar data was obtained between the raw analysis of mPVE vs. bPVE compared to IE, according to the location of the prosthesis.

### 3.6. Middle-Aged Patients (45–65 at the Time of Onset of Prosthetic Surgery)

This sub-analysis included 540 middle-aged patients (45–65 years) at the time of prosthetic surgery, comprising 410 patients with mPVE (75.9%) and 130 patients with bPVE (24.0%). Although the groups were generally similar, the bPVE cohort showed a significantly higher incidence of CAD, HIV, and liver disease, while the mPVE cohort had a higher incidence of preoperative atrial fibrillation. No other baseline differences were noted.

Consistent with the main cohort findings, the onset of bPVE was significantly earlier than mPVE (median 21 months [IQR 5–67] vs. 66 months [IQR 13–139], *p* < 0.001). Unlike the main cohort, there were no statistically significant differences in microbiological data between the mPVE and bPVE groups in this age bracket. Regarding clinical presentation, mPVE patients had a higher incidence of CNS embolism, acute heart failure, and sepsis at presentation. Critically, there were no differences found between the groups concerning surgical indication, the proportion of patients who underwent surgery, hospital mortality, or mortality rate at one year. These data are summarized in Table 5.

## 4. Discussion

PVE affects 20–30% of patients with IE [1,2]. There is a scarce amount of literature that provides a direct comparison between bPVE and mPVE, and even less that analyzes the subgroup of patients aged 45–65 at the time of onset of prosthetic surgery. The present study describes the results of PVE in more than 40 hospitals across Spain gathered in the GAMES-REGISTRY. The main results stemming from the present analysis of the GAMES-REGISTRY data in patients with PVE are the following: 1. bPVE appears earlier than mPVE, including the subgroup of patients aged 45–65 at the time of onset of prosthetic surgery. 2. bPVE and mPVE have some important differences in clinical presentation and paravalvular complications, although there was no difference in surgical indication, and if surgery was performed between both groups. 3. Crude mortality rates were similar between mPVE and bPVE patients, but after adjusting for baseline differences, patients with mPVE were associated with worse outcomes.

One of the most relevant results of our study is the earlier appearance of IA in patients with bPVE, with a median of 13 months after index surgery in the bPVE (p25; p75, 3.5; 45) vs. 50 months in mPVE (p25; p75, 12; 142.5). The latter raised us the following question: What would happen in the subgroup of patients aged 45–65 at the time of onset of prosthetic surgery? These patients could potentially have been selected for both biological and mechanical prostheses. In this age group, patients’ decisions and preferences usually are the main reason to go for one or the other. In this subgroup, bPVE also appeared earlier than mPVE. We hypothesize that biological prostheses, when degenerated, are a substrate for endocarditis. This substate can arise from either prosthetic stenosis, turbulence, platelet aggregation, or other inherent causes of structural valve degeneration. This valve degeneration and the hemodynamic disturbance might be responsible for the early occurrence of IE in this type of prosthetic valve. According to the recent ESC guidelines for the management of IE [13], prosthetic valve selection in IE is influenced by the presence of a woman of childbearing age, advanced age, poor medical compliance, recent ischemic stroke, and evidence of intracranial bleeding. However, with the advent of transcatheter prostheses (TAVI) in recent years, middle-aged patients have tended to prefer biological over mechanical prostheses due to concerns related to the use of anticoagulants and the upsurge of valve-in-valve procedures [14]. Our data may give some insight into the earlier occurrence of IE as an additional factor that could influence the selection of a prosthetic valve. Furthermore, in the literature, there are additional publications analyzing IE on TAVI [12,16] and comparing TAVI-IE with surgical aortic valve replacement (SAVR) IE [11,17,18,19], which highlights the clinical relevance of the information brought by this study.

Regarding IE characteristics, we found a higher frequency of coagulase-negative staphylococci and enterococci among patients with biological prostheses. This is probably due to the older age and comorbidity of these patients, factors classically associated with these microorganisms. Notably, when restricting the analysis to those patients aged 45 to 65 years old, these microbiological differences disappeared. Clinical presentation did not vary between the two groups in certain aspects, including fever, persistent bacteremia, or acute heart failure. However, mPVE had a higher incidence of severe sepsis at hospital admission, IE comprising two valves and prosthetic valve dehiscence. Of the latter, septic shock and IE comprising two valves were two independent risk factors associated with mortality. Understanding these differences in clinical presentation could be important in order to optimize the management of patients with either type of prosthesis.

Generally, bPVE patients should be older and have more comorbidities than those with mPVE; both factors could affect surgical decision-making on whether to operate or not based on the patient’s surgical risk. Remarkably, there was no difference in surgical indication, and surgery was performed in both groups, which highlights the proficiency of the multidisciplinary approach. Regarding postoperative complications, the incidence of de novo postoperative dialysis was similar to that reported in previous publications [20]. On the other hand, the incidence of stroke, pacemaker implantation, postoperative bleeding, and low cardiac output syndrome was noticeably inferior in our study to those reported previously [20].

Importantly, in our analysis, we found that, after adjusting for baseline differences, mPVE was associated with a higher risk of in-hospital and 1-year mortality than bPVE. Additional independent risk factors for mortality were age, Charlson index, SA, IE comprising two valves, central nervous system embolization, and septic shock. These data correlate with previous publications in which advanced age, septic shock, SA, and other comorbidities were found to be independent factors associated with mortality in IE patients [21,22]. In our study, the observed in-hospital mortality rates were similar to those reported in the literature [23,24]. After adjusting for baseline differences, patients with mPVE had higher in-hospital mortality. These findings correlate with a previous meta-analysis on native IE, in which the authors carried out a subgroup analysis on PVE. Their findings were that bPVE showed a lower all-cause mortality in comparison to mPVE, although without statistical significance [25]. On the latter, authors state as a confounding factor that these data do not adjust for age, heart function, and other basic factors [25]. The potentially worse outcomes associated with mPVE could be related to a relatively more aggressive disease in this type of valve, as evidenced in our series in the clinical differences between groups. As stated above, mPVE, although later in occurrence, had significantly higher rates of septic shock, central nervous system embolization, and prosthetic dehiscence. They were also more frequently caused by SA or BGN, two microorganisms associated with acute EI and with higher mortality. This emphasizes the necessity of distinguishing both types of endocarditis, as we demonstrate that mPVE and bPVE have important clinical and prognostic evidence. Only by knowing specific characteristics of both groups can we optimize medical and surgical management of patients and, ultimately, improve their prognosis.

## 5. Limitations

This study is subject to the inherent biases associated with a post hoc analysis of an observational, nationwide database, including potential selection bias related to surgical indications and clinical practice variations across centers. Furthermore, because the database was established in 2008, it lacks contemporary variables such as the EuroSCORE II for surgical risk assessment, and information from advanced imaging modalities like Cardiac Positron Emission Tomography–Computed Tomography (PET-CT) and Cardiac Computed Tomography (CT) was not systematically recorded in the forms. The registry also limited patient follow-up to 12 months, meaning important long-term outcomes, including later reinfections or reoperations, could not be assessed. Lastly, the initial decision regarding the type of prosthesis (biological versus mechanical) was determined by the local surgical team based on existing guidelines and local practice. Although we conducted a specific sub-analysis on middle-aged patients (45 to 65 years) who might have been candidates for either prosthesis type, the influence of additional unmeasured confounding factors cannot be entirely excluded. Despite these methodological limitations, we maintain that our work provides new and meaningful insights into the distinct clinical and microbiological profiles of prosthetic valve endocarditis based on the affected prosthesis type.

## 6. Conclusions

This analysis reveals key distinctions between mechanical and biological PVE. bPVE consistently appears earlier than mPVE, a finding that persisted even in the subgroup analysis of patients aged 45 to 65 years at the time of index surgery. bPVE was characterized by a higher prevalence of healthcare-associated infections and more frequent paravalvular abscess complications. Conversely, mPVE was more frequently caused by *Staphylococcus aureus* and presented with a higher incidence of septic shock and prosthesis dehiscence. Importantly, after adjusting for baseline differences, mPVE was associated with worse clinical outcomes. Recognizing these differential patterns in presentation, microbiology, and prognosis is crucial for optimizing the medical and surgical management strategies for patients with PVE.

## Figures and Tables

**Table 1:** Complete univariate comparison between patients with mechanical vs. biological prosthetic infective endocarditis. AV: Auriculo-ventricular, BMI: Body mass index, bPVE: Biological prosthesis endocarditis, COPD: Chronic obstructive pulmonary disease, ICD: Implantable cardiac defibrillator, IE: Infective endocarditis, mPVE: Mechanical prosthesis endocarditis.

Characteristics and Outcomes: mPVE vs. bPVE			
	Mechanical (733)	Biological (811)	*p*-Value
Baseline data			
Age	66 (59; 73)	75 (69; 79)	<0.001
Sex			
Male	474 (64.6%)	558 (68.8%)	0.085
BMI	25.48 (22.42; 27.76)	25.71 (22.49; 28.72)	0.235
Hypertension	367 (60.9%)	444 (72.4%)	<0.001
Active smoker	49 (8.8%)	27 (4.9%)	0.065
Diabetes	210 (28.6%)	245 (30.2%)	0.502
Hypercholesterolemia	291 (48.2%)	315 (51.4%)	0.474
COPD	133 (18.1%)	151 (18.6%)	0.810
Congestive heart failure	331 (45.1%)	340 (41.9%)	0.201
Coronary artery disease	188 (25.6%)	307 (37.8%)	<0.01
Chronic kidney disease	204 (27.8%)	211 (26.0%)	0.422
Hemodialysis	15 (9.4%)	8 (4.7%)	0.41
Liver disease	49 (6.6%)	56 (6.9%)	0.864
Atrial fibrillation	361 (49.2%)	305 (37.6%)	<0.01
Pacemaker/ICD	113 (18.7%)	83 (13.5%)	0.016
Peripheral artery disease	59 (8.0%)	81 (9.9%)	0.185
Previous stroke	129 (17.5%)	145 (17.8%)	0.886
Immunocompromised (HIV)	3 (0.4%)	7 (0.8%)	0.267
Neoplastic disease	112 (15.2%)	144 (17.7%)	0.191
Intravenous drug addiction	7 (0.9%)	4 (0.4%)	0.281
Previous IE	108 (17.9%)	75 (12.2%)	0.006
Effective antibiotic treatment (days)	42 (25; 49)	42 (25; 50)	0.988
Months after index surgery	56 (12; 151)	19 (4; 55)	<0.01
Charlson index	2 (1; 3)	2 (1; 4)	0.175
Charlson adjusted by age	4 (3; 6)	5 (4; 7)	<0.001
EuroSCORE I log	29.8 (14.8; 52.0)	35.7 (20.7; 60.8)	<0.01
Microbiological data			
*Staphylococcus aureus*	146 (19.9%)	95 (11.7%)	<0.01
Coagulase-negative staph.	192 (26.2%)	266 (32.8)	0.005
*Enterococcus* spp.	98 (13.4%)	144 (17.8%)	0.018
*Streptococcus* spp.	133 (18.1%)	177 (21.8%)	0.071
*Candida* spp.	16 (2.2%)	22 (2.7%)	0.502
Polymicrobial	14 (1.9%)	9 (1.1%)	0.195
Gram-negative bacteria	42 (5.7%)	28 (3.5%)	0.032
Other bacteria	28 (3.8%)	19 (2.3%)	0.092
Community-acquired infection	450 (61.4%)	448 (55.2%)	0.014
Healthcare-related infection	237 (32.3%)	310 (38.2%)	0.016
Dental foci	42 (5.7%)	45 (5.5%)	0.877
Respiratory foci	6 (0.8%)	6 (0.7%)	0.860
Genitourinary foci	22 (3.0%)	40 (4.9%)	0.054
Gastrointestinal foci	52 (7.0%)	70 (8.6%)	0.264
Vascular foci	131 (17.8%)	122 (15.0%)	0.134
Skin foci	38 (5.1%)	40 (4.9%)	0.821
Localization of IE			
Aortic	415 (56.6)	695 (85.7)	<0.01
Mitral	412 (56.2)	188 (23.2)	<0.01
Tricuspid	9 (1.2)	8 (1.0)	0.808
Pulmonary	10 (1.4)	13 (1.6)	0.834
Polivalvular	137 (18.7)	108 (13.3)	0.004
Clinical presentation			
Persistent fever	211 (35%)	207 (33.9%)	0.656
Persistent bacteremia	76 (10.3%)	75 (9.2%)	0.459
Central nervous system embolism	185 (25.2%)	175 (21.5%)	0.089
Peripheral embolism	134 (18.2%)	186 (22.9%)	0.024
New AV conduction disorders	86 (14.3%)	111 (18.3%)	0.131
Acute renal failure	306 (41.7%)	325 (40.0%)	0.504
Acute heart failure	299 (40.7%)	306 (37.7%)	0.219
Severe sepsis	146 (19.9%)	114 (14.0%)	0.002
IE comprising two valves	92 (18.7%)	68 (13.3%)	0.034
Prosthetic valve dehiscence	203 (27.6%)	147 (18.1%)	<0.01
Pseudoaneurysm	74 (10.0%)	84 (10.3%)	0.865
Abscess	229 (31.2%)	303 (37.3%)	0.012
Fistula	32 (4.3%)	41 (5.0%)	0.524
Surgical indication	379 (51.7%)	405 (49.9%)	0.488
Surgery performed	378 (51.6%)	399 (49.3%)	0.377

Frequency (Percent%): *p*-value from chi-square test or Fisher’s exact test when necessary. Median (p25; p75): *p*-value from Mann–Whitney U test.

**Table 2:** Outcomes and postoperative complications.

Postoperative Complications			
	Mechanical (379)	Biological (399)	*p*-Value
Any complication	215 (56.8%)	230 (57.6%)	0.885
Low cardiac output syndrome	66 (17.4%)	66 (28.7%)	0.644
Stroke	6 (1.9%)	9 (3.9%)	0.512
Postoperative bleeding	22 (5.8%)	22 (9.6%)	0.814
Wound infection	46 (21.4%)	39 (17.0%)	0.234
Pneumonia	32 (8.4%)	23 (7.9%)	0.257
Catheter-associated sepsis	8 (2.1%)	9 (3.1%)	0.811
De novo postoperative dialysis	16 (4.2%)	20 (8.7%)	0.628
Pacemaker implantation	16 (4.2%)	18 (7.8%)	0.879

Frequency (Percent%): *p*-value from chi-square test or Fisher’s exact test when necessary.

**Table 3:** Outcomes after mPVE vs. bPVE. bPVE: biological prosthetic endocarditis; IE: infective endocarditis; mPVE: mechanical prosthetic endocarditis.

Outcomes: mPVE vs. bPVE			
	Mechanical (733)	Biological (811)	*p*-Value
In-hospital mortality	238 (32.5%)	243 (30.0%)	0.288
One-year mortality	270 (36.8%)	288 (35.5%)	0.589
Recurrent IE	24 (3.3%)	37 (4.6%)	0.194
Surgery after discharge	23 (3.1%)	21 (2.5%)	0.518

Frequency (Percent%): *p*-value from chi-square test or Fisher’s exact test when necessary.

**Table 4:** Multivariable logistic regression analysis.

Variable	Odds Ratio	95% Confident Interval	*p* Value
Mechanical prosthesis (versus biological)	1.48	1.04–2.11	0.028
Gender (male)	0.76	0.55–1.06	0.109
Age (per year)	1.04	1.02–1.05	<0.001
Charlson Index (per point)	1.10	1.02–1.89	0.015
*Staphylococcus aureus*	1.80	1.13–2.86	0.013
Coagulase-negative staph.	1.40	0.94–2.08	0.096
*Enterococcus* spp.	1.15	0.71–1.86	0.564
Gram-negative bacilli	0.73	0.32–1,64	0.441
Aortic valve implication	1.18	0.80–1.76	0.401
Two or more valve implications	1.47	0.98–2.22	0.072
Dehiscence	1.71	1.23–2.37	0.001
Peripheral embolization	0.92	0.63–1.32	0.644
central nervous system emboli	1.65	1.18–2.32	0.004
Septic shock	5.95	3.91–9.06	<0.001
EuroSCORE I (log, per point)	1.00	0.99–1.00	0.743

Kaplan–Meier survival estimates. Survival at 1 year from diagnosis of PVE. Variables included in the multivariate regression model were age, sex, aortic location, multivalve involvement, Charlson, dehiscence, embolism, septic shock, EuroSCORE I, *S. aureus*, coagulase-negative, enterococcus, Gram-negative bacteria, nosocomial, abscess, and early endocarditis.

**Table 5:** Differences between patients aged 45–65 years during the onset of cardiac surgery.

	Mechanical (410)	Biological (130)	*p*
Baseline data			
Age at onset of IE	65 (59; 70)	63 (59; 66)	0.040
Age at onset of cardiac surgery	57 (51; 62)	59 (56; 63)	0.002
Sex (Male)	273 (66.5%)	102 (78.4%)	0.010
Months after index surgery	69 (17; 150)	31 (8; 83)	<0.01
COPD	74 (18.0%)	25 (19.2%)	0.762
Coronary artery disease	97 (23.7%)	54 (41.5%)	<0.01
Congestive heart failure	191 (46.5%)	57 (43.8%)	0.582
Diabetes	127 (30.9%)	39 (30.0%)	0.834
Immunocompromised (HIV)	1 (0.2%)	3 (2.3%)	0.017
Intravenous drug addiction	3 (0.7%)	2 (1.5%)	0.515
Atrial fibrillation	195 (47.5%)	35 (26.9%)	<0.01
Peripheral artery disease	31 (7.5%)	13 (10.0%)	0.376
Previous stroke	74 (18.0%)	22 (16.9%)	0.770
Neoplasia	65 (15.8%)	23 (17.6%)	0.621
Chronic kidney disease	106 (25.9%)	28 (21.5%)	0.321
Liver disease	29 (7.0%)	19 (14.6%)	0.008
Charlson index	2 (1; 3)	2 (1; 4%)	0.180
Microbiological data			
*Staphylococcus aureus*	85 (20.7%)	25 (19.2%)	0.711
Coagulase-negative Staph.	108 (26.3%)	31 (23.8%)	0.571
*Enterococcus* sp.	56 (13.7%)	17 (13.1%)	0.866
*Streptococcus* sp.	75 (18.3%)	28 (21.5%)	0.412
*Candida* sp.	8 (2.0%)	4 (3.1%)	0.495
Polymicrobial	5 (1.2%)	0	-
Gram-negative bacteria	25 (6.1%)	6 (4.6%)	0.527
Other bacteria	12 (2.9%)	8 (6.2%)	0.090
Community-acquired infection	251 (61.2%)	78 (60.0%)	0.804
Healthcare-related infection	135 (32.9%)	40 (30.8%)	0.647
Dental foci	21 (5.1%)	9 (6.9%)	0.435
Respiratory foci	5 (1.2%)	2 (1.5%)	0.779
Genitourinary foci	11 (2.6%)	5 (3.8%)	0.496
Gastrointestinal foci	38 (9.2%)	12 (9.2%)	0.990
Vascular foci	82 (20.0%)	20 (15.3%)	0.241
Skin foci	20 (4.8%)	6 (4.6%)	0.903
Localization of IE			
Aortic	415 (56.6)	695 (85.7)	<0.01
Mitral	412 (56.2)	188 (23.2)	<0.01
Tricuspid	9 (1.2)	8 (1.0)	0.808
Pulmonary	10 (1.4)	13 (1.6)	0.834
Polivalvular	137 (18.7)	108 (13.3)	0.004
Clinical presentation			
Pseudoaneurysm	40 (9.7%)	11 (8.4%)	0.660
Abscess	130 (31.7%)	52 (40.0%)	0.192
Fistula	21 (5.1%)	10 (7.6%)	0.272
Acute heart failure	172 (41.9%)	41 (31.5%)	0.034
Persistent bacteremia	47 (11.4%)	8 (6.1%)	0.081
Central nervous system embolism	117 (28.5%)	23 (17.6%)	0.014
Peripheral embolism	90 (21.9%)	31 (23.8%)	0.652
Acute renal failure	187 (45.6%)	47 (36.1%)	0.058
Septic shock	77 (18.7%)	15 (11.5%)	0.056
Sepsis	95 (23.1%)	16 (12.3%)	0.008
Surgical indication	221 (53.9%)	73 (56.2%)	0.653
Surgery performed	216 (52.7%)	77 (59.2%)	0.192
Log EuroSCORE I	30.3 (15.9; 51.8)	23.2 (13.4; 42)	0.036
In-hospital mortality	135 (32.9%)	31 (23.8%)	0.051
1-year follow-up mortality	150 (36.6%)	37 (28.5%)	0.09
Frequency (Percent%): *p*-value from chi-square test or Fisher’s exact test when necessary. Median (p25; p75): *p*-value from Mann–Whitney U test.			

BMI: Body mass index, COPD: chronic obstructive pulmonary disease, ICD: implantable cardiac defibrillator, IE: infective endocarditis.

## Data Availability

The data that support the findings of this study are available on request from the corresponding author (de Villarreal-Soto, J.E.). The data are not openly available to preserve the privacy of the participants included in this study.

## Supplementary Materials

The following supporting information can be downloaded at: https://www.mdpi.com/article/10.3390/jcm14248826/s1, Differences in mechanical versus biological endocarditis according to location of the prosthesis.

## Abbreviations

bPVE	Biological prosthetic valve endocarditis
CAD	Coronary artery disease
COPD	Chronic obstructive pulmonary disease
ICD	Implantable cardiac defibrillator
IE	infective endocarditis
LVEF	Left Ventricle Ejection Fraction
mPVE	Mechanical prosthetic valve endocarditis
NYHA	New York Heart Association
PM	Pacemaker
PVE	Prosthetic valve endocarditis
SA	*Staphylococcus aureus*
SAVR	Surgical aortic valve replacement
TAVI	Transcatheter Aortic Valve Implantation
