# Supplementary material for: Clinical and Prognostic Differences Between Mechanical Versus Biological Prosthetic Infective Endocarditis—A Nationwide Database Study
Source: J Clin Med. 2025 Dec 13;14(24):8826. doi: 10.3390/jcm14248826 (PMC12734219; doi:10.3390/jcm14248826)
Supplement: Supplementary file 1 [file jcm-14-08826-s001.zip › jcm-4014233-supplementary.pdf]

Supplementary Materials. Differences in mechanical versus biological endocarditis according to location of the prosthesis

Aortic prosthesis

| | **Mechanical (415)** | **Biological (695)** | **p** |
|---|---|---|---|
| Baseline data | | | |
| Age at onset IE | 66 (58 - 73) | 76 (70 - 80) | <0.01 |
| Age at onset cardiac surgery | 59 (49 - 66) | 73 (68 - 77) | <0.01 |
| Sex (Male) | 326 (78.5) | 495 (71.2) | 0.007 |
| Months after index surgery | 48 (11 - 119) | 18 (4 - 55) | <0.01 |
| COPD | 71 (17.1) | 129 (18.5) | 0.542 |
| Coronary artery disease | 111 (26.7) | 265 (38.1) | <0.01 |
| Congestive heart failure | 166 (40.0) | 274 (39.4) | 0.850 |
| Diabetes | 122 (29.4) | 216 (31.1) | 0.556 |
| Immunocompromised (HIV) | 2 (0.4) | 5 (0.7) | 0.629 |
| Intravenous drug addiction | 5 (1.2) | 1 (0.1) | 0.020 |
| Atrial fibrillation | 160 (38.5) | 244 (35.1) | 0.248 |
| Peripheral artery disease | 40 (9.6) | 73 (10.5) | 0.645 |
| Previous stroke | 58 (13.9) | 121 (17.4) | 0.132 |
| Neoplasia | 72 (17.3) | 121 (17.4) | 0.979 |
| Chronic kidney disease | 109 (26.2) | 181 (26.0) | 0.935 |
| Liver disease | 23 (5.5) | 50 (7.1) | 0.283 |
| Charlson index | 2 (1; 3) | 2 (1; 4%) | 0.177 |
| Microbiological data | | | |
| Staphylococcus aureus | 74 (17.8) | 76 (10.9) | 0.001 |
| Coagulase-negative Staph. | 115 (27.7) | 235 (33.8) | 0.034 |
| Enterococcus sp. | 56 (13.5) | 126 (18.1) | 0.044 |
| Streptococcus sp. | 80 (19.3) | 142 (20.4) | 0.642 |
| Candida sp. | 12 (2.9) | 18 (2.6) | 0.764 |

| | | | |
|---|---|---|---|
| Polymicrobial | 8 (1.9) | 9 (1.3) | 0.027 |
| Gram-negative bacteria | 19 (4.6) | 24 (3.5) | 0.347 |
| Other bacteria | 15 (3.6) | 19 (2.7) | 0.410 |
| Community acquired infection | 261 (62.9) | 377 (54.2) | 0.005 |
| Healthcare-related infection | 128 (30.8) | 274 (39.4) | 0.004 |
| Dental foci | 30 (7.2) | 33 (4.7) | 0.084 |
| Respiratory foci | 4 (0.9) | 5 (0.7) | 0.660 |
| Genitourinary foci | 13 (3.1) | 36 (5.1) | 0.108 |
| Gastrointestinal foci | 28 (6.7) | 59 (8.4) | 0.296 |
| Vascular foci | 69 (16.6) | 106 (15.2) | 0.543 |
| Skin foci | 20 (4.8) | 37 (5.3) | 0.713 |
| Clinical presentation | | | |
| Pseudoaneurysm | 65 (15.6) | 82 (11.7) | 0.066 |
| Abscess | 179 (43.1) | 285 (41.0) | 0.487 |
| Fistula | 29 (6.9) | 40 (5.7) | 0.411 |
| Acute heart failure | 161 (38.7) | 260 (37.4) | 0.645 |
| Persistent bacteremia | 41 (9.8) | 66 (9.4) | 0.834 |
| Central nervous system embolism | 103 (24.8) | 155 (22.3) | 0.337 |
| Peripheral embolism | 77 (18.5) | 167 (24.0) | 0.033 |
| Acute renal failure | 171 (41.2) | 279 (40.1) | 0.728 |
| Septic shock | 64 (15.4) | 70 (10.0) | 0.008 |
| Sepsis | 77 (18.5) | 96 (13.8) | 0.035 |
| Surgical indication | 214 (51.6) | 359 (51.7) | 0.977 |
| Surgery performed | 214 (51.6) | 352 (50.6) | 0.767 |
| Log EuroScore I | 28.5 (14.87 - 52.5) | 35.6 (21.6 - 59.9) | <0.01 |
| In-hospital mortality | 138 (33.3) | 216 (31.1) | 0.452 |
| 1-year follow-up mortality | 155 (37.3) | 255 (36.7) | 0.826 |

Frequency (Percent%): p-value from chi-square test or Fisher exact test when necessary

Median (p25; p75): p-value from Mann-Whitney U test

Mitral prosthesis

| | **Mechanical (412)** | **Biological (188)** | **p** |
|---|---|---|---|
| Baseline data | | | |
| Age at onset IE | 67 (60 - 73) | 75 (68 - 79) | <0.01 |
| Age at onset cardiac surgery | 59 (49 - 66) | 72 (66 - 77) | <0.01 |
| Sex (Male) | 211 (51.2) | 106 (56.3) | 0.239 |
| Months after index surgery | 74 (15 - 174) | 17 (3 - 51) | <0.01 |
| COPD | 75 (18.2) | 36 (19.1) | 0.782 |
| Coronary artery disease | 102 (24.8) | 71 (37.8) | 0.001 |
| Congestive heart failure | 207 (50.2) | 100 (53.1) | 0.503 |
| Diabetes | 113 (27.4) | 54 (28.7) | 0.742 |
| Immunocompromised (HIV) | 2 (0.4) | 3 (1.5) | 0.165 |
| Intravenous drug addiction | 3 (0.7) | 2 (1.0) | 0.675 |
| Atrial fibrillation | 253 (61.4) | 100 (53.1) | 0.058 |
| Peripheral artery disease | 29 (7.0) | 16 (8.5) | 0.525 |
| Previous stroke | 88 (21.3) | 47 (25.0) | 0.322 |
| Neoplasia | 57 (13.8) | 31 (16.4) | 0.394 |
| Chronic kidney disease | 126 (30.6) | 49 (26.1) | 0.259 |
| Liver disease | 32 (7.7) | 10 (5.3) | 0.276 |
| Charlson index | 2 (1 - 4) | 2 (1 - 4) | 0.346 |
| Microbiological data | | | |
| Staphylococcus aureus | 97 (23.5) | 30 (16.0) | 0.035 |
| Coagulase-negative Staph. | 110 (26.7) | 57 (30.3) | 0.359 |

| | | | |
|---|---|---|---|
| Enterococcus sp. | 55 (13.3) | 29 (15.4) | 0.497 |
| Streptococcus sp. | 70 (17.0) | 41 (21.8) | 0.159 |
| Candida sp. | 5 (1.2) | 6 (3.2) | 0.094 |
| Polymicrobial | 6 (1.5) | 0 | - |
| Gram-negative bacteria | 23 (5.6) | 9 (4.8) | 0.688 |
| Other bacteria | 17 (4.1) | 5 (2.7) | 0.485 |
| Community acquired infection | 252 (61.2) | 107 (56.9) | 0.325 |
| Healthcare-related infection | 136 (33.0) | 71 (37.8) | 0.256 |
| Dental foci | 21 (5.0) | 11 (5.8) | 0.703 |
| Respiratory foci | 3 (0.7) | 3 (1.5) | 0.322 |
| Genitourinary foci | 12 (2.9) | 8 (4.2) | 0.395 |
| Gastrointestinal foci | 30 (7.2) | 14 (7.4) | 0.943 |
| Vascular foci | 75 (18.2) | 32 (17.0) | 0.726 |
| Skin foci | 23 (5.5) | 6 (3.1) | 0.205 |
| Clinical presentation | | | |
| Pseudoaneurysm | 24 (5.8) | 9 (4.7) | 0.605 |
| Abscess | 89 (21.6) | 56 (29.7) | 0.030 |
| Fistula | 9 (2.1) | 8 (4.2) | 0.156 |
| Acute heart failure | 185 (44.9) | 78 (41.4) | 0.434 |
| Persistent bacteremia | 43 (10.4) | 16 (8.5) | 0.462 |
| Central nervous system embolism | 113 (27.4) | 47 (25.0) | 0.533 |
| Peripheral embolism | 72 (17.4) | 35 (18.6) | 0.735 |
| Acute renal failure | 182 (44.1) | 74 (39.3) | 0.269 |
| Septic shock | 73 (17.7) | 30 (15.9) | 0.596 |
| Sepsis | 89 (21.6) | 35 (18.6) | 0.402 |
| Surgical indication | 218 (52.9) | 84 (44.7) | 0.061 |
| Surgery performed | 209 (50.9) | 73 (38.8) | 0.004 |
| Log EuroScore I | 31.6 (15.5 - 52.8) | 42.5 (21.8 - 66.0) | <0.01 |

| | | | |
|---|---|---|---|
| In-hospital mortality | 139 (33.7) | 70 (37.2) | 0.404 |
| 1-year follow-up mortality | 161 (39.1) | 82 (43.6) | 0.293 |

Frequency (Percent%): p-value from chi-square test or Fisher exact test when necessary

Median (p25; p75): p-value from Mann-Whitney U test
